# Clinical patterns in asthma based on proximal and distal airway nitric oxide categories

**DOI:** 10.1186/1465-9921-11-47

**Published:** 2010-04-28

**Authors:** James L Puckett, Richard WE Taylor, Szu-Yun Leu, Olga L Guijon, Anna S Aledia, Stanley P Galant, Steven C George

**Affiliations:** 1 Department of Biomedical Engineering, 2420 Engineering Tower, University of California, Irvine, Irvine, CA 92697, USA; 2 Institute for Clinical Translational Science, 1115 Hewitt Hall, University of California, Irvine, Irvine, CA 92697, USA; 3 Children's Hospital of Orange County, 804 W. Collins, Orange, CA 92868, USA; 4 Department of Medicine, 2420 Engineering Tower, University of California, Irvine, Irvine, CA 92697, USA; 5 Department of Chemical Engineering and Materials Science, 2420 Engineering Tower, University of California, Irvine, Irvine, CA 92697, USA

## Abstract

**Background:**

The exhaled nitric oxide (eNO) signal is a marker of inflammation, and can be partitioned into proximal [J'aw_NO _(nl/s), maximum airway flux] and distal contributions [CA_NO _(ppb), distal airway/alveolar NO concentration]. We hypothesized that J'aw_NO _and CA_NO _are selectively elevated in asthmatics, permitting identification of four inflammatory categories with distinct clinical features.

**Methods:**

In 200 consecutive children with asthma, and 21 non-asthmatic, non-atopic controls, we measured baseline spirometry, bronchodilator response, asthma control and morbidity, atopic status, use of inhaled corticosteroids, and eNO at multiple flows (50, 100, and 200 ml/s) in a cross-sectional study design. A trumpet-shaped axial diffusion model of NO exchange was used to characterize J'aw_NO _and CA_NO_.

**Results:**

J'aw_NO _was not correlated with CA_NO_, and thus asthmatic subjects were grouped into four eNO categories based on upper limit thresholds of non-asthmatics for J'aw_NO _(≥ 1.5 nl/s) and CA_NO _(≥ 2.3 ppb): Type I (normal J'aw_NO _and CA_NO_), Type II (elevated J'aw_NO _and normal CA_NO_), Type III (elevated J'aw_NO _and CA_NO_) and Type IV (normal J'aw_NO _and elevated CA_NO_). The rate of inhaled corticosteroid use (lowest in Type III) and atopy (highest in Type II) varied significantly amongst the categories influencing J'aw_NO_, but was not related to CA_NO_, asthma control or morbidity. All categories demonstrated normal to near-normal baseline spirometry; however, only eNO categories with increased CA_NO _(III and IV) had significantly worse asthma control and morbidity when compared to categories I and II.

**Conclusions:**

J'aw_NO _and CA_NO _reveal inflammatory categories in children with asthma that have distinct clinical features including sensitivity to inhaled corticosteroids and atopy. Only categories with increase CA_NO _were related to poor asthma control and morbidity independent of baseline spirometry, bronchodilator response, atopic status, or use of inhaled corticosteroids.

## Background

Asthma is a complex disease characterized by inflammation throughout the respiratory tract from the large airways to the alveoli [1-3]. Moreover, there is mounting evidence that asthma control is correlated with the extent of inflammation, such that symptoms are worse when the inflammation reaches the more peripheral lung compartments [2-6]. One of the great challenges in asthma research is to develop minimally invasive approaches, particularly in children, that could assess the site and extent of inflammation. Such tools could enhance our understanding of the mechanisms that characterize asthma, and prove useful in choosing appropriate therapies [7].

There is mounting data that analysis of exhaled nitric oxide (eNO) could serve as a non-invasive indicator of the extent and site of inflammation in asthma. eNO is a flow dependent signal [8,9], and current guidelines [10] stipulate an exhalation flow of 50 ml/s (FE_NO,50_). FE_NO,50 _is purported to be a marker of airway inflammation, since it is elevated in steroid naïve asthmatics [11], reduced upon administration of anti-inflammatory medications [12], and is correlated with biological [13-15] and physiological [16-18] markers of airway inflammation. However, some patients may fulfill the criteria for the diagnosis of asthma, and yet FE_NO,50 _levels will be normal [19]. Numerous longitudinal studies have investigated the use of FE_NO,50 _as a tool in the clinical management of asthma [20-27]. The results of these studies have been mixed, as four of the studies demonstrated that FE_NO,50 _was of limited use in managing asthmatic symptoms and corticosteroid dose [20,23,24,26]; however, methodological issues in the design of these studies have been raised [28]. Therefore, despite the undeniable link between NO and inflammation, the promise of FE_NO,50 _as a surrogate marker of airway inflammation has yet to be fulfilled, and an alternate approach may be indicated [29].

**Figure 1:**
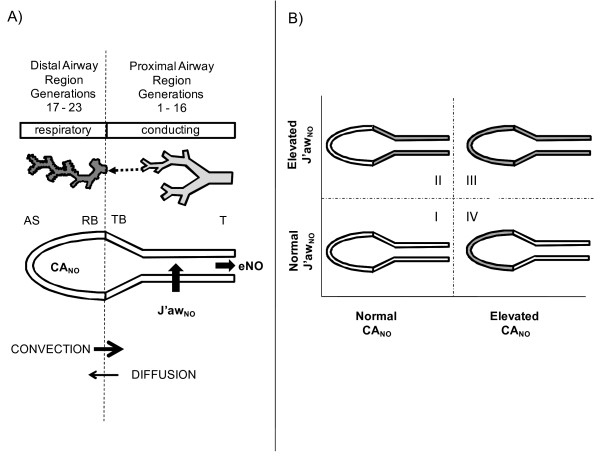
**Schematic of two-compartment model and four eNO categories**. A) During exhalation of nitric oxide (eNO), a steady state mean distal airway/alveolar concentration (CA_NO_, ppb) enters the conducting airway compartment (net transfer is convection minus diffusion) where upon additional NO is transferred from the proximal airway walls (J'aw_NO_, nl/s). CA_NO _represents the respiratory region of the lungs (Weibel generations 17-23). J'aw_NO _represents the larger conducting airway region of the lungs (Weibel generations 1-16), and considers the increasing surface area per unit volume of the airway tree (i.e., trumpet shape). [T: trachea; TB: terminal bronchiole; RB: respiratory bronchiole; AS: alveolar sac]. B) J'aw_NO _and CA_NO _can be selectively elevated (thick gray shading) and may independently characterize proximal and distal lung inflammation, creating four eNO categories: Type I, normal J'aw_NO _and normal CA_NO_; Type II, elevated J'aw_NO _and normal CA_NO_; Type III, elevated J'aw_NO _and elevated CA_NO_; and Type IV, normal J'aw_NO _and elevated CA_NO_.

eNO can be partitioned into proximal airway [J'aw_NO _(nl/s), maximum airway flux, generations 1-16] and distal airway/alveolar contributions [CA_NO _(ppb), alveolar NO concentration, generations 17-23] (Fig. 1A). Increased J'aw_NO _with normal CA_NO _has been reported in adults [30] and children [6] with mild asthma, whereas CA_NO _has been reported to be increased in asthmatics with poor control [5], enhanced symptoms [31], more severe disease [32], and be a predictor of asthma exacerbation in adults [33]. These findings suggest that J'aw_NO _and CA_NO _may be selectively increased and thus independently characterize proximal and distal lung inflammation (Fig. 1B). Hence, we hypothesized that J'aw_NO _and CA_NO _can be selectively elevated in children with asthma, creating four eNO categories, characterized by distinct clinical features.

## Methods

### Study participants

Two hundred pediatric patients with signs and symptoms of asthma within the past three months who presented to the Children's Hospital of Orange County (CHOC) Breathmobile, a mobile asthma clinic staffed with asthma specialists, for an asthma evaluation participated in the cross-sectional study. Criteria for the diagnosis of asthma included a previous history of recurrent coughing, wheezing, shortness of breath (at rest or following exercise), and symptomatic improvement following short acting bronchodilator [1]. Patients were included and considered steroid naïve if they reported no use of inhaled corticosteroid (ICS) in the past 8 weeks, or considered steroid treated if they have reported compliance with prescribed ICS therapy based on NAEPP (National Asthma Education and Prevention Program) guidelines over the previous 8 weeks. Patients were excluded from the study if they had any other heart or lung disease, any smoking within the past five years, or they were ICS treated for less than 8 weeks. Short and long acting β_2 _agonists were withheld for 12 hours prior to the study. Additionally, twenty-one non-asthmatic non-atopic children were enrolled in the study. Each subject and their guardian completed the informed consent documents which had been approved by the University of California, Irvine and CHOC Institutional Review Boards.

### Measurements

Asthma symptoms were quantified using the validated Asthma Control Test (ACT) for children (age 6 - 11 years) [34] and adults (age 12 - 17 years) [35]. Retrospective data on asthma risk factors were collected by the physician. These included severe attacks (exacerbation requiring increased use of albuterol and short-term oral corticosteroids when available), emergency department visits, or hospitalizations within the preceding 8 weeks. Skin prick tests for common allergens (cat, dog, feathers, cockroach, dust mites, mold, weeds, trees and grasses) were performed, and the participant was considered atopic if positive to at least one antigen.

The eNO measurements at multiple flows (50 ml/s, 100 ml/s and 200 ml/s; NIOX Flex, Aerocrine Ltd, Stockholm, Sweden) were performed prior to the pre-bronchodilator spirometric maneuver. eNO measurements at all three flows were performed in triplicate in accordance with ATS guidelines [10], and the order of the nine maneuvers randomized. The physicians did not have access to eNO data at any time; hence clinical management was based solely on history and spirometry.

Standard spirometry was performed (WinDx Spirometer, Creative Biomedics International, CA) in accordance with ATS criteria [36]. To determine the bronchodilator response (BDR), albuterol (180 mcg; 2 puff with spacer) was administered and spirometry was repeated 10 minutes later. The BDR was calculated as the percent change in FEV_1 _following administration of albuterol.

### Calculation of J'aw_NO _and CA_NO_

The average eNO concentration at each flow was calculated following current ATS guidelines [10]. Utilizing a trumpet-shaped model of NO exchange that accounts for axial diffusion [37], we applied a linear least squares analysis to a plot of the average NO elimination rate (product of average exhaled NO and average flow) versus the average exhalation flow to estimate J'aw_NO _and CA_NO _[38].

### Statistical analysis

Spearman rank-order correlation was first evaluated among eNO measurements to evaluate the relationships between FE_NO_, J'aw_NO _and CA_NO_, and the asthmatic subjects were further categorized into four categories based on J'aw_NO _and CA_NO_. Clinical features were compared among the four categories and with the non-asthmatic controls using the Kruskal-Wallis test and the Mantel-Haenszel chi-square test. For variables with significant differences among the groups, paired comparisons were applied with Bonferroni's multiple comparison adjustment. Data are reported using median and range (minimum-maximum), or number of subjects and proportion. Significance level was set at 0.05, and analysis was performed using SAS 9 (Cary, NC).

## Results

In both asthmatic and non-asthmatic populations, 95% of the participants reported an ethnicity of Hispanic. All of the enrolled subjects were able to perform the exhaled NO and spirometric maneuvers. However, among the asthmatic subjects, one was excluded due to missing spirometric data, and twenty were excluded from the analysis since their eNO at multiple exhalation flows did not fit the linear model of NO exchange; this was due to a negative estimated CA_NO _(i.e., non-physiologic interpretation). General features of the entire asthma population are summarized in the first column of Table 1. Our group of asthmatic children was generally mild and well-controlled (median ACT score of 21) with normal baseline spirometry (median FEV_1_/FVC = 87.0).

**Table 1:** Clinical data and risk factors for the exhaled nitric oxide categories

	All asthmatics (n = 179)	Non-asthmatic (n = 21)	Type I *Normal Nitric Oxide* (n = 67)	Type II *Proximal Airway Predominant* (n = 66)	Type III *Proximal and Distal Airway Predominant* (n = 27)	Type IV *Distal Airway Predominant* (n = 19)	Overall test p-value^#^	Paired comparison result^$^
**DEMOGRAPHICS**								
Age	10 (6-17)	10 (6-17)	10 (6-17)	11 (6-17)	11 (6-16)	11 (6-17)	0.13	-----
Gender Male	117 (65%)	12 (57%)	48 (72%)	44 (67%)	16 (59%)	9 (47%)	0.30	-----
Atopy	143 (80%)	0 (0%)	44 (66%)	62 (94%)	23 (89%)	12 (63%)	0.0001	II>I, IV
ICS treatment	110 (61%)	-----	53 (79%)	37 (56%)	5 (18%)	15 (79%)	<0.0001	III<I, II, IV II<I
**EXHALED NITRIC OXIDE**								
FE_NO,50_	19.6 (3.7-186)	8.5 (2.2-15.3)	9.4 (3.7-18.4)	33.4 (17.7-186)	49.9 (22.3-159)	8.5 (4.6-16.9)	0.0001	II, III>NC, I, IV III>II
J'aw_NO _(nl/s)	1.6 (0.1-17)	0.7 (0.1-1.4)	0.7 (0.1-1.5)	3.0 (1.6-17)	3.5 (1.5-13.7)	0.5 (0.1-1.3)	0.0001	II, III>NC, I, IV
CA_NO _(ppb)	1.3 (0.1-13.4)	1.5 (0.1-2.2)	1.0 (0.006-2.3)	0.8 (0.02-2.2)	3.8 (2.4-13.4)	3.1 (2.3-5.1)	0.0001	III, IV>NC, I, II III>IV
**CONTROL and MORBIDITY**								
FEV_1_/FVC	87.0 (70.4-102.1)	89.6 (84.5-102.1)	89.4 (71.7-100)	85.5 (70.4-101)	83.3 (72-100.1)	87.8 (80.7-100.7)	0.0016	III<I, NC
FEV_1 _(%)	106 (67.1-149)	106 (92.6-118)	108 (75.1-149)	105 (67.1-149)	106 (76.2-132)	103 (77.9-127)	0.68	-----
BDR	6.3 (0-35.5) [n = 167]	5.3 (0.6-6.6) [n = 13]	4.1 (0-13.8) [n = 63]	7.0 (0-27.4) [n = 60]	10.1 (0.7-35.5) [n = 26]	5.0 (0.7-10.6) [n = 18]	<0.0001	II>I III>NC, I, IV
ACT	21 (10-27)	-----	23 (17-27)	22 (17-26)	17 (10-23)	17 (10-24)	<0.0001	III, IV<I, II
Morbidity*	26 (15%)	-----	8 (12%)	3 (4.5%)	9 (33%)	6 (32%)	<0.0001	III, IV>II

Data is presented as median (minimum-maximum) or number of subjects (proportion). NC, non-asthmatic control; ICS, inhaled corticosteroid; ACT, Asthma Control Test; BDR, bronchodilator response;

* Number of subjects with at least one event in the past 8 weeks including visits to the emergency department, severe attacks, and hospitalizations.

^#^Mantel-Haenszel chi-square test for Gender, Atopy, ICS treatment and Morbidity; and Kruskal-Wallis test for all other variables. Comparisons made between non-asthmatic control and Type I-IV categories

^$^Bonferroni's multiple comparison adjustment was applied for paired comparison.

### FE_NO,50_, J'aw_NO_, and CA_NO _in non-asthmatic control group

In the non-asthmatic non-atopic children, the median and range of FE_NO,50_, J'aw_NO _and CA_NO _were found to be 8.5 (2.2-15.3), 0.67 (0.13 - 1.44) nl/s, and 1.47 (0.1 - 2.23) ppb, respectively (Table 1). Based on this distribution, and rounding up the maximum value to two significant digits produces a conservative estimate of a threshold for elevated FE_NO,50_, proximal airway NO, and distal airway/alveolar NO: FE_NO,50 _≥ 16 ppb, J'aw_NO _≥ 1.5 nl/s and CA_NO _≥ 2.3 ppb. The results for J'aw_NO _and CA_NO _are similar to the findings of other reports using the two compartment model [39] to partition exhaled NO in non-asthmatic children when axial diffusion is considered [5,40].

### Correlations among FE_NO _and regional nitric oxide parameters

In the asthmatic subjects, J'aw_NO _is strongly correlated with FE_NO,50 _(r = 0.99), FE_NO,100 _(r = 0.93) and FE_NO,200 _(r = 0.95). CA_NO _is not correlated with FE_NO,50 _(r = 0.09) or FE_NO,100 _(r = 0.11). At the highest flow of 200 ml/s, the contribution of the proximal airways is reduced, but CA_NO _is only very weakly correlated (r = 0.23) with FE_NO,200_.

### Exhaled nitric oxide (eNO) categories

**Figure 2:**
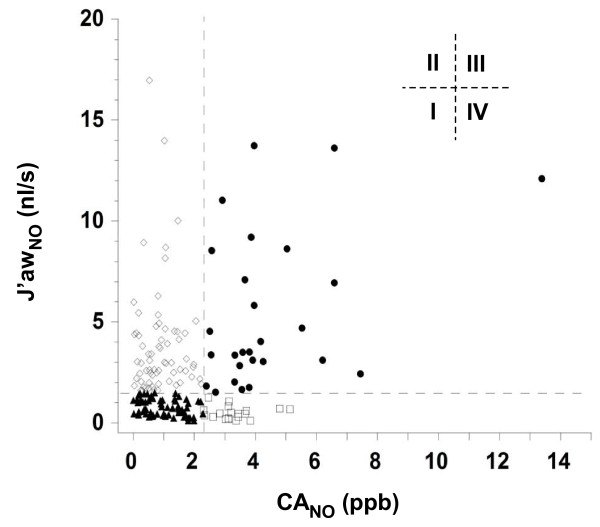
**Exhaled nitric oxide (eNO) categories**. Scatter plot between maximum proximal airway nitric oxide flux (J'aw_NO_) and distal airway/alveolar nitric oxide concentration (CA_NO_). When axial diffusion is considered, there is no correlation (Spearman rank, r = -0.002) between J'aw_NO _and CA_NO_. Asthmatic eNO categories based on thresholds for non-asthmatics J'aw_NO_(≤ 1.5 nl/s) and CA_NO _(≤ 2.3 ppb): Type I (white triangle), normal J'aw_NO _and normal CA_NO_; Type II (white diamond), elevated J'aw_NO _and normal CA_NO_; Type III (white circle), elevated J'aw_NO _and elevated CA_NO_; and Type IV (white square), normal J'aw_NO _and elevated CA_NO_.

CA_NO _and J'aw_NO _are not correlated (r = -0.002) indicating that they provide independent information. Hence, asthmatic subjects were classified into four eNO categories (Fig. 2) based on the upper limit of non-asthmatic thresholds for J'aw_NO _(≥ 1.5 nl/s) and CA_NO _(≥ 2.3 ppb):

**Type I: **J'aw_NO _and CA_NO _not elevated; n = 67, (37%)

**Type II: **elevated J'aw_NO_; n = 66, (37%)

**Type III: **elevated J'aw_NO _and elevated CA_NO_; n = 27, (15%)

**Type IV: **elevated CA_NO_; n = 19, (11%)

There were no gender or age differences amongst the four eNO categories and the non-asthmatic controls (Table 1). The proportion of atopic subjects was significantly different between the groups (p = 0.0001, Table 1), where Type II had significantly higher rates compared to Type I and Type IV.

Based on patient history, inhaled corticosteroid (ICS) naïve was defined as no oral or ICS within the last 8 weeks and ICS treated was defined as prescribed ICS treatment for at least 8 weeks. Significant differences (p < 0.0001, Table 1) existed between the eNO categories with respect to ICS use. The proportion of subjects who were ICS treated was significantly less in Type III when compared to the other eNO categories and was significantly less in Type II compared to Type I.

As defined above, J'aw_NO _was elevated in Type II [3.0 (1.6-17.0) nl/s] and Type III [3.5 (1.5-13.7) nl/s] but not in non-asthmatic controls [0.7 (0.1-1.4) nl/s], Type I [0.7 (0.1-1.5) nl/s] and Type IV [0.5 (0.1-1.3) nl/s]. There was no difference in J'aw_NO _between Type II and Type III. Details are summarized in Table 1.

Also, by design, CA_NO _was elevated in Type III [3.8 (2.4-13.4) ppb] and Type IV [3.1 (2.3-5.1) ppb] but not in non-asthmatic controls [1.5 (0.1-2.2) ppb], Type I [1.0 (0.006-2.3) ppb] and Type II [0.8 (0.02-2.2) ppb] (Table 1). Furthermore, CA_NO _was found to be significantly (p = 0.02) greater in Type III when compared to Type IV.

Since FE_NO,50 _was strongly correlated with J'aw_NO_, FE_NO,50 _was significantly elevated in Type II [33.4 (17.7-186.2) ppb] and Type III [49.9 (22.3-158.9) ppb] when compared to non-asthmatic controls [8.5 (2.2-15.3) ppb], Type I [9.4 (3.7-18.4) ppb] and Type IV [8.5 (4.6-16.9) ppb] (Table 1). However, it was also found that FE_NO,50 _was significantly higher in Type III than Type II.

### Clinical patterns of eNO categories

There were significant differences in FEV_1_/FVC ratio (p = 0.0016), where Type III [83.3 (72-100.1)] was lower than non-asthmatic controls [89.6 (84.5-102.1)] and Type I [89.4 (71.7-100)]. However, the median FEV_1_/FVC for Type III was within normal limits based on NHLBI/NAEPP guidelines [1]. With regards to FEV_1_/FVC < 80%, there were approximately 4%, 18%, 22% and 0% for Types I to IV, respectively. There were no significant differences in FEV_1 _(% predicted) amongst the four eNO categories and the non-asthmatic controls (Table 1).

The BDR was performed in 180 subjects and was found to be significantly different (p < 0.0001, Table 1) amongst the five groups. The BDR in Type III was found to be significantly greater than non-asthmatic controls, Type I, and Type IV. Furthermore, the BDR in Type II was significantly greater than Type I.

The ACT score was significantly different (p < 0.0001, Table 1) amongst the eNO categories. The ACT scores in Type III and Type IV were significantly lower compared to Type I and Type II. Over 80% of Type I and Type II had an ACT score > 19, indicative of good asthma control; while 78% of Type III and 90% of Type IV had and ACT score ≤ 19, indicative of poor asthma control.

The retrospective asthma risk factors (severe attacks, emergency department visits, and hospitalizations) were defined as any event during the last eight weeks (Table 1). There were significant differences amongst the eNO categories in the proportion of children with at least one asthma morbidity event (p < 0.0001, Table 1). Multiple comparison adjustment demonstrated that the frequency of morbidity events was significantly greater in Type III and Type IV when compared to Type II.

## Discussion

Our study has demonstrated that proximal airway (J'aw_NO_) and distal airway/alveolar NO (CA_NO_) are not correlated in children with generally mild well-controlled asthma, thus allowing identification of four distinct eNO categories: Type I (normal nitric oxide), Type II (proximal airway predominant), Type III (proximal and distal airway predominant) and Type IV (distal airway predominant). Our main finding is that eNO categories with increased CA_NO _(i.e., Type III and Type IV) have much worse asthma control and morbidity (Table 1), despite different rates of ICS treatment, atopy, baseline spirometry, and BDR. Together, these findings support the presence of distinct inflammatory categories based on regional eNO, and suggest that distal NO (CA_NO_) may be a more clinically sensitive, objective measure of asthma control compared to spirometry and proximal NO (FE_NO,50_, J'aw_NO_).

### Inflammatory categories in asthma

Traditionally, asthma has been grouped into two categories on the basis of etiology [41]: intrinsic and extrinsic. More recently, management guidelines have stratified asthma on the basis of severity [1]. However, the severity classification scheme does not consider the degree of airway inflammation, a factor which could improve clinical management [42,43]. There are only a few reports which utilize inflammatory patterns to characterize adult asthma [44-46] and none to the best of our knowledge, describe inflammatory categories in pediatric asthma. The idea of stratifying asthma based on inflammatory patterns was first presented by Wenzel et al. in 1999 [44]; severe asthmatics were divided into two subtypes based on the presence or absence of eosinophils in the bronchial biopsy specimen. In a 2006 study by Simpson et al. [45], the asthmatics were segregated into four inflammatory subtypes using induced sputum eosinophil and neutrophils counts. More recently, a 2009 study by Nadif et al. [46] demonstrated that blood eosinophil and neutrophil counts can be used to characterize adult asthma. However, these methods are more invasive in nature, more challenging to perform in children, and may not be ideally suited for the serial monitoring of asthmatic subjects. The current data suggest a relatively simple approach for non-invasively gauging information about the location and extent of inflammation in the asthmatic lung.

A general summary of the eNO categories is presented in Table 2. Well-controlled asthmatics generally appear in Type I and Type II categories. The common features between these categories are the absence of distal airway/alveolar inflammation (low CA_NO_) and normal baseline spirometry, despite differences in proximal NO (FE_NO,50 _and J'aw_NO_), ICS treatment, atopy, and BDR. In contrast, poorly controlled asthmatics tend to fall into Type III and Type IV categories. The common features between these categories are the presence of distal airway/alveolar inflammation (high CA_NO_), despite differences in proximal NO (FE_NO,50 _and J'aw_NO_), ICS treatment, and BDR. Thus, distal inflammation, as indicated by an elevated CA_NO_, appears to be the most robust predictor of asthma control in our group of relatively mild asthmatic children relative to traditional indices such as baseline spirometry and ICS therapy.

**Table 2:** Clinical patterns of the eNO categories

	*Type I* *Normal Nitric Oxide*	*Type II* *Proximal Airway Predominant*	*Type III* *Proximal and Distal Airway Predominant*	*Type IV* *Distal Airway Predominant*
**EXHALED NITRIC OXIDE**				

J'aw_NO _(nl/s)	< 1.5	≥ 1.5	≥ 1.5	< 1.5

CA_NO _(ppb)	< 2.3	< 2.3	≥ 2.3	≥ 2.3

FE_NO,50 _(ppb)**^#^**	▪	▪▪▪▪	▪▪▪▪	▪

**THERAPY AND ATOPY**				

Atopy	▪▪▪	▪▪▪▪	▪▪▪▪	▪▪▪

ICS treatment	▪▪▪▪	▪▪▪	▪	▪▪▪▪

**LUNG FUNCTION**				

Abnormal Spirometry	▪	▪	▪	▪

BDR > 10%	▪	▪▪	▪▪▪	▪

**CONTROL and RISK**				

ACT ≤ 19	▪	▪	▪▪▪▪	▪▪▪▪

Morbidity*	▪	▪	▪▪	▪▪

Proportion of subjects: ▪▪▪▪ ≥ 75%, ▪▪▪ 50-74.9%, ▪▪, 25-49.9%; ▪ <25%.

^#^Abnormal FE_NO,50 _is based on the upper limit of the non-asthmatic, non-atopic controls (≥ 16 ppb).

Abnormal spirometry: FEV_1 _% predicted < 80% or FVC % predicted < 80% or FEV_1_/FVC < 80%.

Asthma control test (ACT) ≤ 19 is indicative of poor asthma control.

* Proportion of subjects with at least one event in the past 8 weeks including visits to the emergency department, severe attacks, and hospitalizations.

### Proximal versus distal airway inflammation

Our observation that J'aw_NO _is strongly related to FE_NO,50 _indicates that an exhalation flow of 50 ml/s is low enough such that FE_NO,50 _is dominated by the proximal airway contribution, and partitioning the exhaled NO signal to estimate J'aw_NO _provides no additional information. Therefore, to characterize the inflammatory status of the proximal lung FE_NO,50 _is sufficient. However, even though the distal airway/alveolar concentration is a greater fraction of FE_NO _at higher flows, it remains very small, and FE_NO,200 _is only very weakly correlated with CA_NO_; hence, the exhaled NO signal must be partitioned using a mathematical model to estimate the much smaller concentration of CA_NO_. Our observation that J'aw_NO _and CA_NO _are not correlated indicates they provide independent information regarding region specific NO. Of note is the confounding role of axial diffusion of NO which has been demonstrated in both healthy adults [37,47-49] and those with stable asthma)[50], but not in active asthma; if axial diffusion of NO is not considered in our data set, then a strong positive relationship (r = 0.71) is observed between J'aw_NO _and CA_NO_. In other words, a significant proximal airway source can contaminate the distal airway/alveolar region via axial (or "back") diffusion leading to an artificial elevation in CA_NO _and an erroneous positive correlation.

Proximal airway NO (FE_NO,50 _and J'aw_NO_) has not been consistently associated with asthma control or a predictor of exacerbation [20-23]. One possibility for these findings is the confounding influences of ICS therapy and atopy, both of which strongly impact proximal airway NO [5,12,51-53]. For example, Paraskakis et. al [5] has demonstrated that FE_NO,50 _is no different between asthmatic and atopic non-asthmatic children. In our categories, FE_NO,50 _(and J'aw_NO_) progressively increased between Types I, II, and III (9.4, 33.4, 49.9 ppb) as the proportion of subjects on ICS decreased (79%, 56%, 18%), respectively. Type IV asthmatics do not follow this trend (79% and 8.5 ppb, respectively). This observation is likely due to the peripheral (small airway/alveolar) nature of the inflammation which is not targeted by ICS.

In contrast, several different groups have reported clinically relevant observations regarding CA_NO _and asthma control [5,6,31-33]. For example, increased levels of CA_NO _have been reported in asthmatics with nocturnal symptoms [31], asthmatics with poor control (based on bronchodilator use) [5] or refractory to ICS treatment [32], as well as a predictor of asthma exacerbation [33]. These observations are consistent with our results of poor control in children with elevated CA_NO _(Types III and IV comprising 26% of our population). The association between poor asthma control and elevated CA_NO _is likely due to the presence of inflammation in the distal lung which can contribute to airflow limitation [2-4]. However, our data, as well as data in the literature, also demonstrate that that CA_NO _is much less dependent on confounding influences of ICS therapy and atopy compared to J'aw_NO _or FE_NO,50_. Paraskakis et. al [5] studied a group of more severe steroid-treated asthmatic children, and reported that CA_NO _was elevated in the children with poorly controlled asthma, but not in atopic non-asthmatic controls. Although our patient population represented relatively mild asthmatic children, we observed that the rate of atopy and ICS therapy differed amongst the eNO categories, but neither was related to asthma control (e.g., rate of atopy was highest in the well-controlled Type II and rate of ICS highest in poorly controlled Type IV and well-controlled Type I).

Although ICS therapy does not appear to impact CA_NO_, nor improve control in asthmatic children with elevated CA_NO_, reports in the literature demonstrate that CA_NO _and asthma control do respond to systemic medications, namely oral corticosteroids [32,54] and leukotriene receptor antagonists [55]. Together, these results suggest that proximal airway and distal airway/alveolar NO respond differently to treatment, and thus may be of clinical utility [29]. However, well-designed longitudinal studies are necessary to determine appropriate therapeutic options for achieving asthma control in children with elevated CA_NO_.

### Study limitations

Our pediatric population was predominately Hispanic (95%). Ethnicity may impact response to asthma medications due to genetic differences in cellular receptors [56], and thus our results may not apply to other ethnic groups. Approximately 10% of the asthmatic subjects did not fit the two compartment model of NO exchange in the lungs. However, the model was successfully applied in all of the non-asthmatic subjects. These results are similar to the findings of Paraskakis et al. [5], and may be related to heterogeneous ventilation and inflammation patterns in some asthmatic subjects [57]. Finally, we enrolled subjects independent of inhaled corticosteroid use which allowed us to evaluate the differential effect of ICS therapy on proximal and distal eNO and the relationship to asthma control.

## Conclusions

In summary, we propose a novel method for non-invasively categorizing asthma based on region specific NO parameters J'aw_NO _and CA_NO_. We have shown that J'aw_NO _and CA_NO _can be selectively elevated in asthma, permitting identification of four eNO categories with distinct clinical patterns. The categories characterized by increased CA_NO _(Types III and IV) have much worse (but similar to each other) asthma control and morbidity, despite significantly different rates of ICS therapy, atopy, baseline spirometry and BDR. Hence, our preliminary observations in a mobile asthma clinic setting, suggest that categorizing asthma using proximal and distal NO may be clinically useful, both in terms of feasibility and management of asthma control. For example, our data suggest that Type III and Type IV asthmatics may not achieve good asthma control with standard ICS therapy. Future studies must address the dynamic (i.e., longitudinal) nature of the categories with regards to therapy and disease progression, the relationship to other inflammatory indices such as cells in the sputum, blood or bronchial biopsy, whether this categorization strategy is relevant in maintaining asthma control and reducing asthma morbidity, and the role of axial diffusion of NO in active asthma. Nonetheless, these results suggest that not one (i.e., FE_NO,50_), but all aspects of eNO may be important in the management of asthma, and that partitioning eNO to determine CA_NO_may significantly improve the clinical relevance of the eNO signal.

## Competing interests

The authors declare that they have no competing interests.

## Authors' contributions

JP assisted in the design of the experiment, data collection,, and the writing the manuscript. RT assisted in the data analysis and interpretation. S-YL performed the statistical analysis, and assisted in the interpretation of the data and the writing of specific sections of the manuscript. OG assisted in the collection and interpretation of the data. AA assisted in the data collection through the completion of the IRB approval, and also assisted in the interpretation of the data. SPG and SCG conceived of the orginal concept and experimental design, and assisted in the data interpretation and writing of the manuscript. All authors read and approved the final manuscript.
